# AggreProt: a web server for predicting and engineering aggregation prone regions in proteins

**DOI:** 10.1093/nar/gkae420

**Published:** 2024-05-27

**Authors:** Joan Planas-Iglesias, Simeon Borko, Jan Swiatkowski, Matej Elias, Martin Havlasek, Ondrej Salamon, Ekaterina Grakova, Antonín Kunka, Tomas Martinovic, Jiri Damborsky, Jan Martinovic, David Bednar

**Affiliations:** Loschmidt Laboratories, Department of Experimental Biology and RECETOX, Faculty of Science, Masaryk University, Brno, Czech Republic; International Clinical Research Center, St. Anne's University Hospital Brno, Brno, Czech Republic; Loschmidt Laboratories, Department of Experimental Biology and RECETOX, Faculty of Science, Masaryk University, Brno, Czech Republic; International Clinical Research Center, St. Anne's University Hospital Brno, Brno, Czech Republic; IT4Innovations, VSB – Technical University of Ostrava, 17. listopadu 2172/15, 708 00 Ostrava-Poruba, Czech Republic; IT4Innovations, VSB – Technical University of Ostrava, 17. listopadu 2172/15, 708 00 Ostrava-Poruba, Czech Republic; Loschmidt Laboratories, Department of Experimental Biology and RECETOX, Faculty of Science, Masaryk University, Brno, Czech Republic; International Clinical Research Center, St. Anne's University Hospital Brno, Brno, Czech Republic; IT4Innovations, VSB – Technical University of Ostrava, 17. listopadu 2172/15, 708 00 Ostrava-Poruba, Czech Republic; IT4Innovations, VSB – Technical University of Ostrava, 17. listopadu 2172/15, 708 00 Ostrava-Poruba, Czech Republic; Loschmidt Laboratories, Department of Experimental Biology and RECETOX, Faculty of Science, Masaryk University, Brno, Czech Republic; International Clinical Research Center, St. Anne's University Hospital Brno, Brno, Czech Republic; IT4Innovations, VSB – Technical University of Ostrava, 17. listopadu 2172/15, 708 00 Ostrava-Poruba, Czech Republic; Loschmidt Laboratories, Department of Experimental Biology and RECETOX, Faculty of Science, Masaryk University, Brno, Czech Republic; International Clinical Research Center, St. Anne's University Hospital Brno, Brno, Czech Republic; IT4Innovations, VSB – Technical University of Ostrava, 17. listopadu 2172/15, 708 00 Ostrava-Poruba, Czech Republic; Loschmidt Laboratories, Department of Experimental Biology and RECETOX, Faculty of Science, Masaryk University, Brno, Czech Republic; International Clinical Research Center, St. Anne's University Hospital Brno, Brno, Czech Republic

## Abstract

Recombinant proteins play pivotal roles in numerous applications including industrial biocatalysts or therapeutics. Despite the recent progress in computational protein structure prediction, protein solubility and reduced aggregation propensity remain challenging attributes to design. Identification of aggregation-prone regions is essential for understanding misfolding diseases or designing efficient protein-based technologies, and as such has a great socio-economic impact. Here, we introduce AggreProt, a user-friendly webserver that automatically exploits an ensemble of deep neural networks to predict aggregation-prone regions (APRs) in protein sequences. Trained on experimentally evaluated hexapeptides, AggreProt compares to or outperforms state-of-the-art algorithms on two independent benchmark datasets. The server provides per-residue aggregation profiles along with information on solvent accessibility and transmembrane propensity within an intuitive interface with interactive sequence and structure viewers for comprehensive analysis. We demonstrate AggreProt efficacy in predicting differential aggregation behaviours in proteins on several use cases, which emphasize its potential for guiding protein engineering strategies towards decreased aggregation propensity and improved solubility. The webserver is freely available and accessible at https://loschmidt.chemi.muni.cz/aggreprot/.

## Graphical Abstract

**Graphical Abstract:**
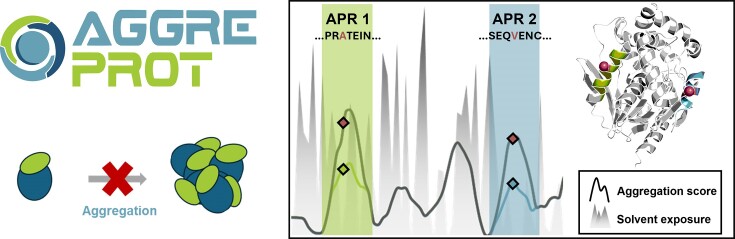

## Introduction

Recombinant proteins, versatile in applications from industrial biocatalysts to therapeutics, benefit from the remarkable progress in computational prediction of protein structures (1,2). Despite accurately and rapidly predicting protein structures (3–5), new computational algorithms provide a single conformation corresponding to the global minimum of the free energy landscape without assuming anything regarding the underlying folding pathways (6,7). *In vivo*, protein folding is primarily driven by the burial of hydrophobic residues whose exposure can lead to non-native self-association, misfolding and, ultimately, aggregation. The formation of such misfolded aggregates is triggered by various factors (8) and is associated with severe pathologies such as Alzheimer's or Parkinson's diseases (9). Additionally, binding sites, oligomerization interfaces, or other surface residues important for protein function *in vivo* can often act as potential APRs outside the native environment of proteins, e.g. during recombinant production in host organisms, or in a buffer in their purified form. Consequently, formation of inclusion bodies, low yields, and aggregation/precipitation of purified proteins are commonly encountered nuisances during protein production. Fast and accurate identification of APRs is therefore crucial for mitigating these issues and improving the efficiency of recombinant protein technologies. Among aggregates, amyloids represent a specific class characterized by highly organized two-dimensional structures. Amyloids are formed by stacked repetitive units of protein molecules stabilized by an intermolecular network of hydrogen bonds within their cross-β-sheet architecture (10) which, however, often adopt different morphologies (11,12). Nonetheless, they share a common structural kernel which is believed to be an important driver of amyloid formation and essential for its stability (13–15). These regions (APRs) are therefore perfect targets for designing mutations that decrease aggregation propensity and, consequently, improve protein solubility.

Several algorithms have been designed to address aggregation challenges. Depending on the type of input data they accept, such algorithms are classified as sequential or structural predictors. Sequential or linear predictors are typically fast, and rely either on experimentally derived scales of aggregation propensity, like in AGGRESCAN (16) or CamSol intrinsic (17), or features based on the protein amino acid composition (frequency distribution, physico-chemical properties, β-sheet propensity, hydrogen-bond optimal packing density), like in Waltz (18), Tango (19), PASTA (20), SALSA (21) or FoldAmyloid (22). Structural methods require a three-dimensional protein structure as an input, exploit structural features such as solvent accessibility or analysis of interactions, and are consequently slower. Their major advantage is the ability to identify structural APRs formed by sequentially distant residues which are impossible to detect by the linear predictors. An example of a structural predictor is AGGRESCAN 3D (23), which also considers protein dynamics in the aggregation prediction. The algorithms from both classes greatly contributed to our understanding of protein aggregation and solubility on a molecular level, and are being frequently used to identify APRs in proteins with varying degrees of success.

Over the past several years, a third generation of predictors emerged based on machine learning approaches such as support vector machine in the Budapest Amyloid Predictor (24), random forest classifiers in RF Amyloid (25) and Amylogram (26), and many others (27) including ANuPP (28), FishAmyloid (29) or CORDAX (30). The availability of computational power and advances in the ML field make the development of this class of predictors highly appealing. However, discerning molecular features important for aggregation is difficult due to the convoluted nature of the underlying calculations and therefore, the true impact of these predictors is yet to be seen based on the successful use cases, which are so far scarce. One of the main problems in engaging the prediction of APRs in proteins is the lack of training and reference data. Currently, WaltzDB (31), a database containing 1416 annotated hexapeptides entries (as of February 2024), including 515 and 901 amyloid and non-amyloid forming peptides, respectively, is the most comprehensive dataset for training aggregation predictors. Their aggregation propensity has been verified using several experimental techniques, including secondary structure determination, amyloid-specific dye binding, and electron microscopy imaging. Moreover, each hexapeptide entry includes annotations on several calculated energetical parameters from FoldX (32) and secondary structure prediction and architectural zipper class predicted by CORDAX (30). In contrast, two other curated databases, AmyPro (33) and CPAD 2.0 (34), compile protein sequences with annotated APRs based on the literature search and are widely used for predictor benchmarking. However, we and other groups have recently contested this relatively common practice by experimentally investigating the amyloid propensity of some of the sequences and revealed many inaccuracies in the database labels (30,35). As a result, comparing protein-level predictions of aggregation propensity between different algorithms based on these databases is potentially biased, and their performance should always be validated by an experiment. Due to the scarcity of training data, the machine learning-based predictors are often hexapeptide-centred, making it difficult to get a profile view of the complete protein. Furthermore, those methods often lack contextual information such as three-dimensional structure, prediction of solvent accessibility, or transmembrane propensity, which need to be taken into account for a successful protein engineering campaign (35–37).

To provide the community with an easy-to-use predictor of protein aggregation and engineering tool, we have developed a sequence-based, user-friendly webserver for predicting APRs in proteins, AggreProt (https://loschmidt.chemi.muni.cz/aggreprot/). The server features an ensemble of five deep neural networks (DNNs) that output a single per-residue aggregation profile for up to three possible input protein sequences. Additionally, our server provides information on the solvent-accessible area and transmembrane propensity to aid users in distinguishing potential surface-exposed APRs from hydrophobic cores. The graphical interface features an interactive sequence and structure viewer, allowing the comparison of profiles from multiple proteins at once. Trained on a hexapeptides set with experimentally evaluated aggregation propensities, AggreProt outperforms or is comparable to state-of-the-art algorithms during benchmarking on training (WaltzDB) and validation (AmyPro) datasets. We successfully applied our predictor to solubilise the aggregating haloalkane dehalogenase LinB (35) and poorly soluble luciferase from *Amphiura filiformis* (in preparation). Here we illustrate how AggreProt can pinpoint differential aggregation behaviours in several proteins annotated in the recently released *in-house* database SoluProtMut^DB^ (38).

## Materials and methods

### Datasets

Two different datasets were used to train, validate, and test the deep neural networks (DNNs) that are at the core of AggreProt. First, WaltzDB (31) contains approximately 1400 hexapeptides, of which nearly 500 are labelled as amyloid prone and the rest (cca. 900) are not. WaltzDB was randomly split into training and testing datasets. 90% of the stratified contents of the database (keeping the label ratio) were used to train and optimise the parameters of our DNNs during their training and hyperparameter search process (WaltzDB-training). The remaining 10% was kept for testing (WaltzDB-testing). The second data set employed in testing was AmyPro (33). It consists of the sequences of 162 amyloid proteins with their amyloid-prone regions (APRs) annotated. Only 37 of such proteins do not enclose any hexapeptide present in WaltzDB (AmyPro37). Among these, 10 correspond to proteins with very long (> 50 amino acids) APRs annotated. The remaining 27 proteins (conforming AmyPro27 dataset, Supplementary File S1) were used to test the performance of our DNNs in the domain of whole protein sequences.

### Metrics

The performance of the DNNs was assessed with standard metrics derived from the confusion matrix. Considering prediction and ground truth (experimental APRs), *True Positives (TP)* are defined as ground truth positives (amyloid prone) predicted as positives; *False Positives (FP)* as ground truth negatives (non-amyloid prone) predicted as positives; by opposition, ground truth positives predicted as negatives define the set of *False Negatives (FN);* and finally, ground truth negatives predicted as negatives conform the *True Negatives (TN)*. From these definitions, the following are derived:


(1) \begin{equation*}{\mathrm{Precision }}\left( {{\mathrm{Prec}}} \right) = {\mathrm{TP}}/\left( {{\mathrm{TP}} + {\mathrm{FP}}} \right)\end{equation*}

(2) \begin{equation*}{\mathrm{Recall }}\left( {{\mathrm{Rec}}} \right) = {\mathrm{TP}}/\left( {{\mathrm{TP}} + {\mathrm{FN}}} \right)\end{equation*}

(3) \begin{equation*}{\mathrm{Specificity }}\left( {{\mathrm{Spec}}} \right) = {\mathrm{TN}}/\left( {{\mathrm{FP}} + {\mathrm{TN}}} \right)\end{equation*}

The performance of the networks was assessed as the area under (Au) the Receiver Operating Characteristic (Recall versus 1 − specificity) and Precision/Recall curves (ROCC and PRC respectively). On ROCCs, Youden's *J* statistic (39) was used to determine the optimal threshold for prediction.

### Network features and hyperparameter search

Our DNNs predict the aggregation propensity of an input hexapeptide sequence, considering the atomic feature description of each amino acid in the hexapeptide as employed in ANuPP (28). Thus, the first input layer dimension (number of neurons) corresponds to the number of atomic features, 36 per each residue in the hexapeptide. During the training procedure, the architecture and internal parameters of the networks were optimized. To this end, WaltzDB-training was used in a 5-fold cross-validation fashion. The optimised parameters and the search range are summarized in Table 1.

**Table 1.** Details of network optimization

Optimized parameter	Value range
Number of bidirectional layers	[1,2]
Number of dense layers	[1,2,3]
Dropout values	[0–0.8], in 0.2 step range
Number of neurons per layer	[8–512], in powers of 2 step range
Batch size	[16, 32]
Learning rate	[10^−4^–10^−1^], in powers of 10 step range

A total of 841 hyperparameter combinations were examined, each resulting network evaluated in terms of AuROCC. The top performing architecture, summarized in Table 2, provided five individual predictors, one for each data split in the 5-fold cross-validation procedure. The models were obtained at the training epoch where the training and validation ROC curves diverged, to avoid overfitting (see Results). The final AggreProt predictor is thus an ensemble of five DNNs (consensus values aggregated across the ensemble by average). Each of the networks is formed by an architecture composed of eight layers. All the training procedure was performed using a custom codebase developed in the scope of this work, based on the TensorFlow Framework (40).

**Table 2.** Architecture of AggreProt deep neural networks (DNNs)

Layer type	Input neurons	Output neurons	Layer no.
Input	36*6	64*6	1
Bidirectional (LSTM)	64*6	64	2
Bidirectional (LSTM)	64	96	3
Dense + dropout	96	32	4–5
Dense + dropout	32	32	6–7
Dense	32	1	8

LSTM stands for long short-term memory.

### Deep neural networks testing

The remaining 10% of hexapeptides not used during the training procedure (WaltzDB-testing) were used to test the performance of the resulting networks. The performance was evaluated in terms of the area under the ROC curve (AuROCC). To identify amyloid-prone regions (APRs) in the complete sequence of proteins, protein sequences were fragmented into overlapping hexapeptides using a six-amino-acid- windows shifted one amino acid at a time. Each of the resulting hexapeptides was then evaluated by the DNN ensemble, and the results were aggregated (averaged) per residue using a sliding window procedure mirroring the previous one. Protein sequences in the AmyPro27 dataset were evaluated by this procedure, and the performance of the network was assessed in terms of area under the ROC and PR curves (AuROCC and AuPRC, respectively). Finally, the Segment OVerlap score (SOV), as defined by Rost and co-workers (41) and used in ANuPP (28), was also used to evaluate the correctness of the predictions in a whole sequence context. The SOV score can be calculated both for APR and non-APR segments. Other state-of-the-art predictors that base their predictions on the sole input of sequence, namely Waltz (18), Tango (19), ANuPP (28) and AGGRESCAN (16) were also evaluated using AmyPro27 and the aforementioned metrics. For the servers that limit the length of the input, several requests were made per each protein in AmyPro27 (Waltz and Tango), and their results were co-catenated.

### Prediction of protein structure, transmembrane propensity, and solvent accessible surface area. Alignment of sequences and three-dimensional (3D) visualization

The input of protein structure is optional; hence, when only sequence is provided AggreProt fetches the closest sequence from AlphaFoldDB (42) using BLAST (43), provided that the *E*-value of the hit from AlphaFoldDB is at maximum 1e10^−80^. Transmembrane (TM) propensity is predicted from sequence using TMHMM 2.0 (44). The Solvent Accessible Surface Area (SASA) is calculated only if a structure is provided or obtained from AlphaFoldDB, and the values are calculated by DSSP (45), using the SBI library (46). Sequences are aligned using the Needleman and Wunch alignment algorithm from BioJava (47). The 3D view pane implements Mol* (48).

## Results

### Performance on hexapeptides

The performance of AggreProt DNNs expectedly varied depending on the data used during the training procedure. While on the training set the AuROCC could reach 100%, it never exceeded 90% on the validation set which provided us with the overfitting limits of the predictor. We selected the training epoch where the training and validation ROC curves diverged as the final predictive models to avoid overfitting (see Methods, Supplementary Figure S1A). The best DNN ensemble achieved an AuROCC of 88.7% (Supplementary Figure S1B) on the independent WalztDB-test set.

### Performance on whole protein sequences and comparison to the state-of-the-art methods

In comparison to predicting the aggregation propensity of isolated hexapeptides, working the whole protein sequence requires further processing. First, the input sequence needs to be converted into a collection of overlapping hexapeptides. When the hexapeptide-level predictions are done, the results should be aggregated to the residue level. For validation purposes, we selected 27 sequences from AmyPro (AmyPro27) obtained after (i) filtering to remove overlaps with the training dataset (37/160), and (ii) removal of sequences containing long APRs (>50 aa, 10/37) mostly corresponding to low complexity, prion-like domains (49–51). Using AmyPro27, we compared the prediction from AggreProt to those obtained by four different sequence- or ML-based state-of-the-art methods, namely Waltz (18), Tango (19), ANuPP (28), and AGGRESCAN (16). We observe a great disparity of AuROCC and AuPRC values for AggreProt performance, (see Supplementary File S2). The average performance values across the dataset reach 0.64 and 0.32 in terms of AuROCC and AuPRC, respectively. It must be noted that, if proteins with long APRs (larger than 50 amino acids long) are considered (AmyPro37) these values drop to 0.53 and 0.23, respectively. This indicates that if such long APRs correspond to relevant biological events (e.g. low complexity domains, LCDs), then the chances of detecting them by our hexapeptide-trained predictor are lower. We have recently described and experimentally verified that this drop in statistics is, at least partly, due to miss-annotations in the validation dataset (35). Our findings were in agreement with those of Louros and co-authors (30), and stress the correspondence of AggreProt predictions at hexapeptide or whole-protein levels on experimental data (35). Nonetheless, the predictor can still resolve short (between 5 and 10) residues stretches within the long-annotated APRs that often overlap with the amyloid aggregation kernels found within the cores (13–15). In comparison, other sequence-based state-of-the-art methods do not perform significantly better in our blind independent testing set. Whether on residue-level or SOV validation, AggreProt reached similar or better evaluation performance compared to other sequence-based methods (Table 3). The only notable exceptions are PASTA (20)and SALSA (21), for which their training sets significantly overlap with our validation AmyPro27 one (13 and 15 out of 33 training proteins, respectively).

**Table 3.** Evaluation of different sequence-based aggregation propensity predictors on AmyPro27

Predictor	AuROCC	AuPRC	SOV APR	SOV Non-APR	Reference
AggreProt	**0.64**	0.32	**54.8**	41.7	This study
Waltz	0.57	0.24	30.5	56.0	(18)
Tango	**0.64**	**0.33**	52.4	**61.5**	(19)
ANuPP	**0.64**	0.27	**54.8**	57.3	(28)
Aggrescan	***0.64***	***0.33***	54.7	47.0	(16)
Fold Amyloid	0.62	0.30	50.1	47.7	
PASTA*	*0.72*	*0.38*	*7.14*	*47.1*	(20)
SALSA*	*0.67*	*0.35*	*5.3*	*14.9*	
Camsol	0.36	0.16	16.3	19.1	
Aggrescan3D^§^	*0.57*	*0.26*	*71.5*	*17.8*	

The top-performing predictors in each category are shown in boldface. AuROCC and AuPRC stand for area under the Receiver Operating Characteristic and Precision Recall curves, respectively. SOV stands for Segment OVerlap score (41), which can be calculated over aggregation prone regions (APR) or non-aggregation prone regions (Non-APR). *PASTA and SALSA performance is biased since a large portion of their training sets overlaps with our AmyPro27 validation set (13 and 15 out of 33 proteins, respectively). ^§^Aggrescan3D belongs to a different family of predictors, those that consider the structure of the protein as an input.

We extended our comparison to an example of structure-based methods, Aggrescan3D (23), which overall achieves similar results in all metrics except in overlapping true APRs, where it stands over all other compared predictors. Finally, to test AggreProt performance on sequences with low complexity, we further analysed set of 10 proteins containing LCDs or prion-like domains (PrLDs) and compared the results with sequence regions known to form amyloids (Supplementary Note 1, Supplementary Figure S2).

### Experimental validation

**Figure 1.**
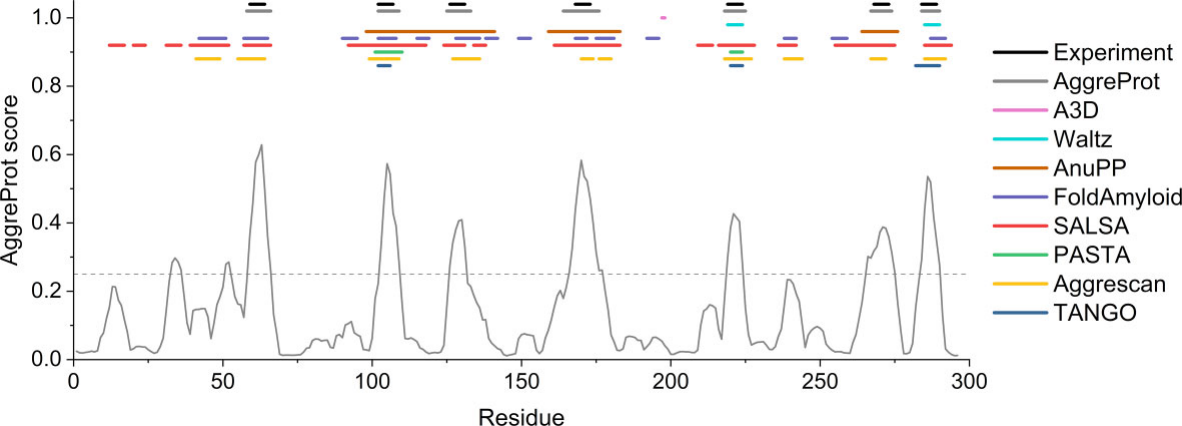
Experimental validation of AggreProt and comparison to other methods. APRs in LinB as previously determined in (35) (Experiment) or determined by predictors. The APR annotations were made using the default settings of each predictor according to the authors’ recommendations. Even though the two peaks in AggreProt prediction around residues 35 and 50 reached the cut-off, their length was too short (less than six residues) to be considered as APRs.

A recurring problem in the protein amyloid aggregation domain is the validity of the standard datasets used (30). To face this problem, we engaged a systematic experimental validation on 37 hexapeptides and 13 mutational variants of a model haloalkane dehalogenase LinB (HLD LinB). Our experiments showed AggreProt accuracy in predicting APRs: 30 out of 37 hexapeptides were correctly predicted with 2 further cases being inconclusive. Moreover, AggreProt correctly identified APRs in HLD LinB and was used to guide the design of mutations that decreased aggregation propensity and increased yield of soluble protein (the best mutations by 100%). The ability of AggreProt and other predictors to detect APRs in LinB is shown in Figure 1. We also purposely introduced mutation into the buried APR which led to significantly compromised structural integrity of the protein, to further highlight the importance of SASA information during design (35). Finally, we employed AggreProt to detect APRs in an insoluble luciferase from *Amphiura filiformis*. Subsequent engineering of one of the detected regions leads to solubilization of the protein (in preparation). These extensive experimental validations together with the herein presented predictions on three different proteins demonstrate the usefulness of AggreProt in identifying and engineering APRs in proteins.

## Web server usage

### AggreProt workflow

**Figure 2.**
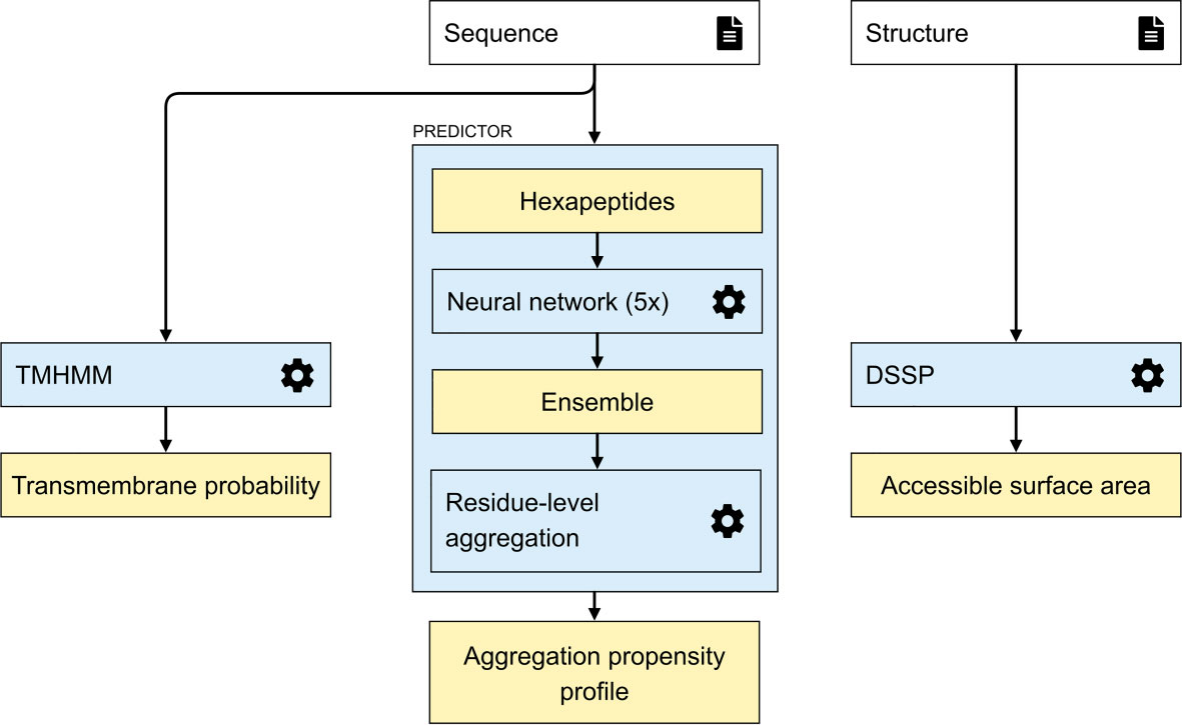
AggreProt schematic workflow. The different calculations on provided input types (sequence and structure) are depicted in sequential order. The input sequence is used to generate the TM and aggregation propensity profiles. The input structure is required to generate SASA profiles. Inputs (compulsory and optional) are indicated by a ‘file’ icon. Processes (third party software and internal processing) are indicated by a cog wheel and blue boxes. Outputs (partial and final) are indicated in yellow.

AggreProt web server has been designed to provide the users with an easy experience when identifying APRs in their input sequence. The server combines its dedicated amyloid aggregation propensity predictor described above with transmembrane (TM) propensity and solvent accessible surface area (SASA) calculations that provide structural context for the analysed protein sequence. The overall workflow of the operations in the server is shown in Figure 2.

### Data input

First, the user inputs their protein sequence(s) in FASTA format, which are quickly scanned for integrity, i.e. that headers and sequence are in place. Currently, the webserver allows to upload up to three different sequences as input for posterior comparison. Then, depending on the number of input sequences, an input box to upload a structure file associated to each of them appears dynamically. In this step the structure can be uploaded from a custom file (PDB and mmCIF formats are accepted) or can be downloaded from RSCB PDB. In case the user cannot provide an input structure, AggreProt offers the option to fetch it from AlphaFoldDB (42) either using AlaFoldDB ID, searching directly on the external database, or performing a BLAST search (43) on it (this latter option being significantly slow). Optional job title and e-mail address can be given for easy identification of the job submitted and posterior notification of results completion, respectively. However, these two pieces of information are not required by the server, which can be used freely by all users without any login requirement.

### Results output

**Figure 3.**
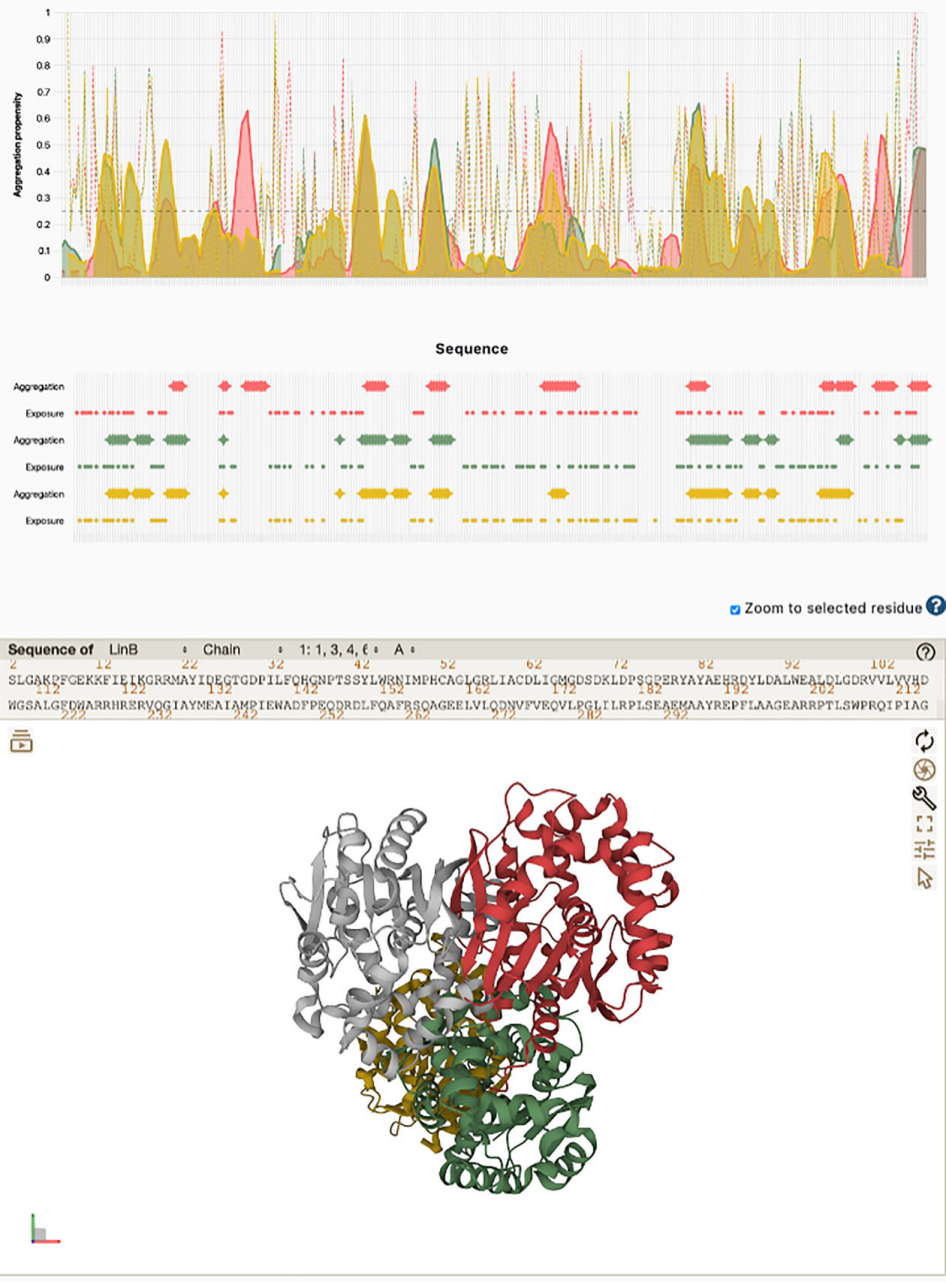
AggreProt results page. Propensity and 3D panes are displayed on top of each other and interactively linked. The propensity pane allows setting the cut-off threshold value for aggregation propensity and to display the results for any of the input proteins at will. The different propensity series are colour-coded identifying the input protein and differently dashed to identify each of the series (aggregation, TM, and SASA). Below the propensity chart, the protein sequences and the predictions in a glyph-encoded view are provided. At the bottom, the 3D view pane which is bidirectionally interactive with the rest of the elements of the results page. The figure displays three haloalkane dehalogenases: LinB (red), DhaA wild type (green) and DhaA115 (hyper-stable mutant, yellow) (54).

After the job submission, the user is redirected to a page displaying a summary of the submitted job where its progression can be tracked. The job summary indicates the job ID and name (if provided), the submission time stamp, and the status of the job. Upon calculation completion, the job status becomes ‘DONE’, and the calculated results are made available. On the top, a profile visualization window displays the obtained results in two different graphical forms: (i) as aligned profiles (central part, propensity chart) and (ii) as a glyph annotation (bottom, sequence display) (Figure 3, top panel). The profiles and the glyphs are colour coded so that the results for individual input proteins (if more than one was submitted) can be easily compared.

The aggregation propensity profile is shown in a semi-transparent solid hue, while TM propensity and SASA are indicated by dotted and dashed lines, respectively. Within this section of the visualizer (propensity chart), hovering over any sequence position, renders additional information about the protein residue and the individual prediction values for each of the propensities calculated. The corresponding glyphs are shown for each propensity series (amyloid aggregation, TM and SASA) according to their own thresholds. These glyphs help detecting continuous stretches of protein sequence that are predicted to be in a membrane or exposed. At the top of the visualization pane, the aggregation propensity threshold can be adjusted (by default it displays the threshold associated with the Youden J statistic for the predictor), and the display of the different input proteins can be adjusted independently. Increasing the threshold results in decreasing sensitivity (number of peaks detected) and increasing specificity (number of true peaks detected); decreasing the threshold achieves the opposite. The thresholds for TM (0.4) and SASA (0.25) are predetermined based on previous experience (52,53). Below, an adjustable slide ranger tool allows to zoom in on a particular section in the protein sequence(s) or to zoom out to a wider protein sequence range.

Linked to the profile visualization pane the server provides a three-dimensional (3D) representation of the input proteins (Figure 3, bottom panel) if provided by the user or otherwise fetched from the databases. The 3D view pane is interactively connected to the profile visualizer, such as when clicking an element (i.e. one amino-acid position) in one of the panes, the corresponding selection is also highlighted. In this way, from the upper visualizer the amino-acids conforming an APR (represented by a peak or by a continuous stretch of glyphs in the Aggregation series) can be selected and then highlighted for inspection in the 3D panel. The interactive behaviour also works vice-versa: selecting a region of interest from the structure will result in that region highlighted in the sequence profile visualizer, where the user can easily check what is the aggregation propensity of the selected region. At the very bottom of the results page, an executive help section indicating the user how to navigate the profile and the 3D visualizations.

### Use cases

In our previous study we showed that AggreProt can correctly identify APRs that form amyloid fibrils as isolated peptides and promote aggregation within the context of the model protein HLD LinB (35). Furthermore, we show that mutations in these APRs decrease aggregation propensity and simultaneously increase solubility, providing a higher yield of the model protein. Here, we use the observed link between aggregation and solubility and extend it further to three model cases. Specifically, we validate the functionality of the presented webserver by the analysis of the deep scanning mutational data from two proteins: (i) Type III polyketide synthase and (ii) TEM β-lactamase (52,53) from SoluProtMut^DB^ (38). SoluProtMut^DB^ is a manually curated database containing effects of mutations on protein solubility. Singularly it contains data on deep mutational scanning experiments, which can be exploited to perform a retrospective experiment: do solubilizing mutations correspond to the regions predicted by AggreProt as APRs? Are the effects of such mutations correctly estimated by AggreProt? Here, we illustrate that despite being trained on amyloid forming hexapeptides, our predictor can also detect APRs that in the correct context and with appropriately designed mutations may increase solubility. The aggregation propensities of significantly solubilized protein variants were compared to the respective wild types using AggreProt webserver. We use this reverse engineering approach to test how AggreProt can successfully guide the protein engineering strategies to reduce the aggregation and increase solubility. By exploring two of the proteins with deep mutational data, we showcase AggreProt capabilities and demonstrate the potential pitfalls and complexity of such protein solubilizing campaigns.

**Figure 4.**
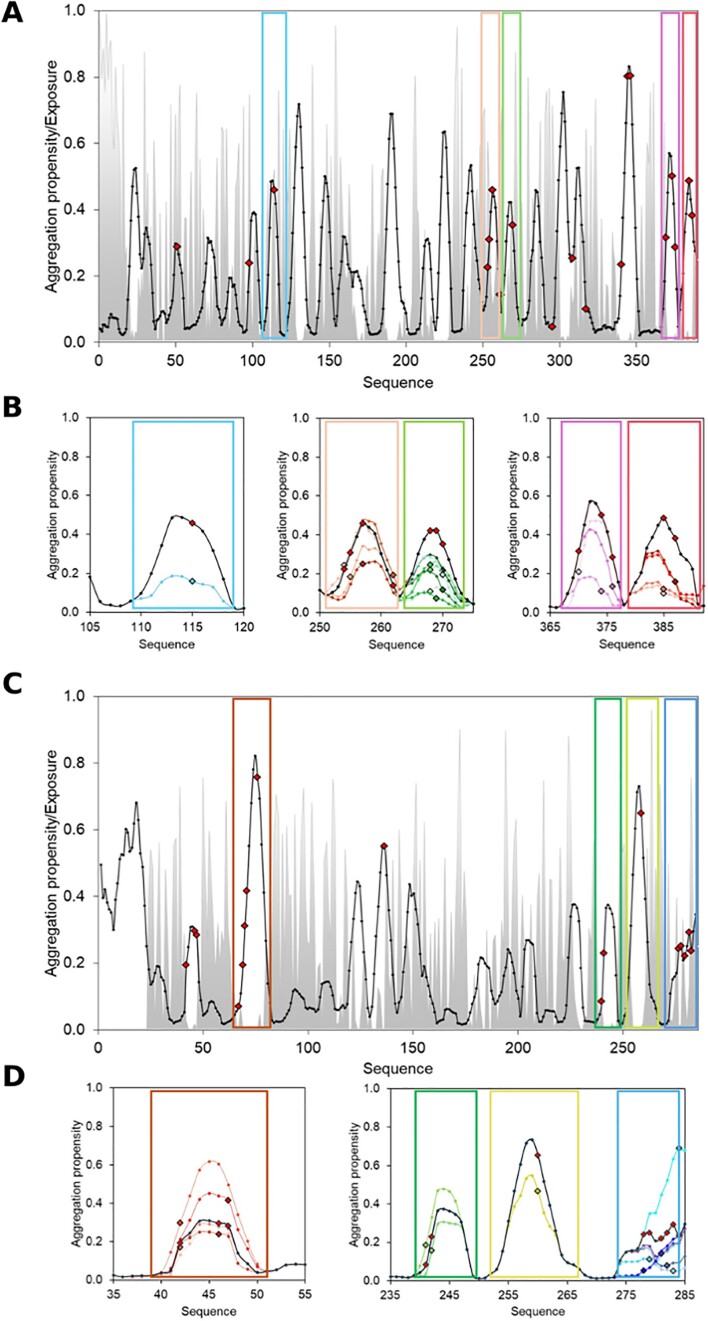
The overview of the predicted mutational effect of selected Type III polyketide synthase (**A, B**) and TEM-1.1 β-lactamase (**C, D**) variants. (A, C) The wild-type aggregation profile (solid line) is shown in the context of solubilizing mutations occurring in predicted APRs (red diamonds), other solubilizing mutations outside predicted peaks (white diamonds), predicted SASA (dotted line, greyed out area), and aggregation propensity threshold (dashed horizontal line). (B, D) The predicted effect of the mutation is shown for some selected exposed APRs (different colour hues); mutated residues are shown as coloured diamonds, red for the wt-profile, and matching the hue of the mutation for the mutational profiles.

The significantly solubilizing mutations marked as manually curated from SoluProtMut^DB^ were selected for all three proteins. Different thresholds of the solubility change were used to yield a similar number of mutants for all proteins (≥2.0 for Type III polyketide synthase and ≥7.0 for TEM β-lactamase and Levoglucosan kinase). Mutations on Type III polyketide synthase showed a strikingly high correspondence with the detected peaks by AggreProt. The effect of such mutations as predicted by AggreProt agreed with the recorded experimental effect in the vast majority of cases (Figure 4, Supplementary Table S1).

For TEM β-lactamase, the solubilizing mutations were colocalized in regions with high aggregation propensity that were both exposed and buried. The solubilizing mutations found in the exposed APRs followed the same logic that we followed in our previous work (35): replacing hydrophobic residues with more polar or charged ones. On the contrary, mutations found in buried regions consisted in the increase of local hydrophobicity, and our predictor failed to recognize them as solubilizing (Figure 4, Supplementary Table S2). This observation agrees with the practice of leaving hydrophobic buried regions out of engineering campaigns (55) and designing only solvent-accessible amino acids.

### Type III polyketide synthase (Uniprot ID: A0A3G4RHW3)

In total, 31 mutations (56) were selected based on the filters described above. Interestingly, 28 of those (90%) were in the regions detected as APRs by AggreProt, (Figure 4, Supplementary Table S1). The effect of these mutations was subsequently evaluated as the changes in the AggreProt profile (Figure 4, Supplementary Table S1). The peaks in APR regions were reduced in 21 cases (75%) suggesting solubility improvement, in five cases (18%) the mutations did not affect the size of the peaks in APR regions suggesting minor effects, and only in two cases (7%) the corresponding peaks were in APR regions increased indicating changes towards lower solubility. Overall, these results confirm that AggreProt can identify aggregation-prone regions with high confidence and that the effect of a large majority of substitutions introduced into these regions are correctly predicted by AggreProt.

### TEM-1.1 (β-lactamase, Uniprot ID: P62593)

In total, 59 out of the 80 mutations (74%) selected based on our criteria were in the predicted APRs. The subsequent analysis (Figure 4, Supplementary Table S3) showed that AggreProt predicted a reduction of the aggregation peak in 34 cases (58%), thus lowering predicted aggregation propensity, while in 25 cases (42%) the effect was neutral or negative, indicating higher predicted aggregation propensity. However, in the case of β-lactamase the solubilizing mutations corresponded to buried APRs, and the solubilizing effect estimated by AggreProt depended on the type of mutation. Mutations increasing local hydrophobicity are predicted by AggreProt to increase the aggregation propensity. While this is often the case in exposed APRs, it is not a conserved trend in buried ones. In a protein engineering campaign, the consideration of such contextual information is therefore crucial not to disrupt the hydrophobic core of the protein (55). The comprehensive presentation of aggregation-promoting residues together with their location within protein structure provides essential information for rational protein engineering using AggreProt.

### Nb.b201 nanobody (PDB ID: 5vnw, chain C)

An additional use case showing the performance of AggreProt on Nb.b201 nanobody and experimentally validated solubilising mutations (57) is presented in Supplementary materials (see Supplementary Note 2 and Supplementary Figure S3).

## Discussion and outlook

AggreProt web server presents the community with an easy-to-use application for predicting and engineering amyloid aggregation prone regions in proteins based on their sequence. Its performance on standard validation datasets is in par or better than the state-of-the-art sequence-based methods: Waltz (18), Tango (19), ANuPP (28), AGGRESCAN (16), PASTA (20), SALSA (21), CamSol intrinsic (17), and FoldAmyloid (22). Also, for reference, the comparison was extended to one widely-used structure-based method, Aggrescan3D (23). In comparison to those, AggreProt does not present any limitation in the input sequence length, providing a residue-level prediction for the whole protein sequence. Our tool also facilitates the interpretation of results allowing the user to compare up to three different input proteins in one single run. Moreover, it provides useful contextual information in the form of transmembrane propensity and solvent accessible surface area, whenever possible. Our previous experience indicate that this feature is crucial for successful design of aggregation-reducing mutations into proteins. AggreProt predictor has been experimentally validated: (i) at hexapeptide level, with correct predictions in 30 out of 37 cases, and (ii) in two protein solubilizing campaigns. On the first campaign, AggreProt detected 4 solvent-accessible APRs on haloalkane dehalogenase LinB; each of the APRs was re-designed resulting in a more soluble protein (35). The second, on a luciferase from *Amphiura filiformis* also detected the correct region that, upon design, resulted in increased solubility (in preparation).

We illustrate the application of our webserver in the retrospective experiment based on deep mutational scanning data on two different proteins: type III polyketide synthase and TEM β-lactamase (58). We extracted the most solubilizing mutations on each of these proteins from SoluProtMut^DB^ (38), and mapped them to the aggregation profile obtained by AggreProt. In the first example, we found a perfect overlap between the solubilizing mutations and the predicted APRs Moreover the solubilizing effect of these mutations was recapitulated by the AggreProt predictions. In the case of TEM β-lactamase, most of the solubilizing mutations corresponded to predicted APRs, but happened on buried regions of the protein. Here, the capacity of AggreProt to correctly predict the mutation behaviour depended on the nature of the mutation: we had correct (solubilizing) estimations for hydrophobic-to-hydrophilic mutations and wrong predictions otherwise. This illustrates the power of the contextual information since understanding the solvent accessibility of the amino acid to be mutated is crucial to correctly predict the effect of the mutation. We further illustrate the performance of AggreProt in an additional biomedically and biotechnologically relevant test example of a nanobody. There our predictor was able to correctly identify the mutational effect of 6 out of 8 solubilized variants.

AggreProt as presented is a powerful resource to identify APRs in proteins, based only on their sequence input. However, the final goal of reducing the protein aggregation propensity can only be achieved by engineering its sequence. We will further develop AggreProt and provide the user with a ‘Design’ panel, where the engineering campaign could be outlined, and its outcomes predicted. We will facilitate this task in future versions of our tool, by implementing the following strategies: First, enabling the user to fine-tune the boundaries of detected APRs and define custom ones. Correctly defining the APRs boundaries is crucial for implementing one of the trending strategies in solubilizing amyloid prone proteins developed by the Switch Lab: mutating the gatekeeper residues lying on the APR boundaries (59). Second, implementing two different mutational strategies: (i) targeting the aforementioned gatekeepers and subsisting residue each pair by any of the 5 residues arginine, lysine, aspartic acid, glutamic acid and proline (for a total of 25 variants per APR), and ii) targeting the exposed residue closer to the peak of the APR and saturating its position (for a total of 19 variants per APR). In our vision, the user will have the ability to choose and mix-and-match among the strategies and redefine the residues to be mutated. To present the user with a final solution, first all selected mutations will be evaluated by AggreProt and their new profiles super-imposed to the original one. Then, after the user selects one mutation per APR, an ideal multi-mutation sequence will be compiled, evaluated by the predictor, and finally presented to the user for inspection.

## Supplementary Material

gkae420_Supplemental_Files

## Data Availability

Aggreprot is freely available and accessible at https://loschmidt.chemi.muni.cz/aggreprot/.

## Notes

Present address: Antonín Kunka, Protein Biophysics Group, Department of Biotechnology and Biomedicine, Technical University of Denmark, Søltofts Plads, Building 227, 2800, Kgs. Lyngby, Denmark.
