# Supplementary material for: AggreProt: a web server for predicting and engineering aggregation prone regions in proteins
Source: Nucleic Acids Res. 2024 May 27;52(W1):W159–69. doi: 10.1093/nar/gkae420 (PMC11223854; doi:10.1093/nar/gkae420)
Supplement: gkae420_Supplemental_Files [file gkae420_supplemental_files.zip › AggreProt_1917_NAR_R1_SI_FINAL.pdf]

AggreProt: A Web Server for Predicting and Engineering Aggregation Prone Regions in Proteins

Joan Planas-Iglesias[1,2,§], Simeon Borko[1,2,§], Jan Swiatkowski[3,§], Matej Elias[3], Martin Havlasek[1,2], Ondrej Salamon[3], Ekaterina Grakova[3], Antonín Kunka[1,2,#], Tomas Martinovic[3], Jiri Damborsky[1,2], Jan Martinovic[3,*] and David Bednar[1,2,*]

[1] Loschmidt Laboratories, Department of Experimental Biology and RECETOX, Faculty of Science, Masaryk University, Brno, Czech Republic
[2] International Clinical Research Center, St. Anne's University Hospital Brno, Brno, Czech Republic
[3] IT4Innovations, VSB – Technical University of Ostrava, 17. listopadu 2172/15, 708 00 Ostrava-Poruba, Czech Republic

[§]The authors wish it to be known that, in their opinion, the first three authors should be regarded as Joint First Authors.

[#]The current affiliation of the Author is: Protein Biophysics Group, Department of Biotechnology and Biomedicine, Technical University of Denmark, Søltofts Plads, Building 227, 2800, Kgs. Lyngby, Denmark

* To whom correspondence should be addressed. Tel: +420605143394; Email: 222755@mail.muni.cz. Tel: +420597329598; Email: jan.martinovic@vsb.cz

Supplementary materials

SUPPLEMENTARY NOTE 1: PERFORMANCE ON LOW COMPLEXITY OR PRION-LIKE DOMAINS. 2

SUPPLEMENTARY NOTE 2: ADDITIONAL USE CASE. 2

SUPPLEMENTARY FIGURE S1 3

SUPPLEMENTARY FIGURE S2 4

SUPPLEMENTARY FIGURE S3: 6

SUPPLEMENTARY TABLE S1 7

SUPPLEMENTARY TABLE S2 8

SUPPLEMENTARY FILE 1. AMYPRO27 9

SUPPLEMENTARY FILE 2. VALIDATION OF AGGREPROT ON AMYPRO27 9

SUPPLEMENTARY FILES 3-11. VALIDATION OF OTHER PREDICTORS ON AMYPRO27 9

REFERENCES 9

Supplementary Note 1: Performance on low complexity or prion-like domains.

To test AggreProt performance on sequences with low complexity, we further analysed set of 10 proteins containing low complexity domains (LCDs) or prion-like domains (PrLDs) and compared the results with sequence regions known to form amyloids (**Supplementary Figure S2**). LCDs and PrLDs are composed by a limited number of amino acids compared to the balanced distribution in the rest of the proteome (1), and are often linked to diseases (e.g. FUS, hnRNPA, or TDP43 in amyotrophic lateral sclerosis (2)). AggreProt identified APRs in 8 out of 16 amyloid forming sequences found within PrLDs and LCDs (green, **Supplementary Figure S2**), and detected increased aggregation propensity for at least 3 additional ones (yellow, **Supplementary Figure S2**). In comparison, SALSA was the only algorithm from the other seven tested that had a similar level of success (8 out of 16 hits). The aggregation scores of LCDs were generally low compared to the APRs in folded domains, which reflects the underrepresentation of such sequences in the training dataset. Despite this, AggreProt could still detect local increase in aggregation propensity in some of them (e.g., FUS, **Supplementary Figure S2**). This suggests that although not optimal, AggreProt can still be useful in detecting short APRs within LCDs or prions based on the closer inspection of aggregation profiles (i.e., peaks below given threshold) and prior knowledge regarding the sequence composition or protein domain organization. Since AggreProt is not suited for identification of LCDs and PrLDs, we recommend using other algorithms designed for that purpose (10.1093/nar/gkaa339).

Supplementary Note 2: Additional Use Case.

Nb.b201 nanobody (PDB ID: 5vnw, chain C)

The recently engineered nanobody *Nb.b201* (3) was analyzed using AggreProt webserver (**Supplementary Figure S3**) to verify its capabilities of predicting the mutational effect on the solubility and aggregation of biomedically relevant targets such as antibodies. In the original study, in total 7 mutations were designed and combined into 12 potentially solubilized variants. Subsequent cross-interaction chromatography (CIC) measurements confirmed the solubilizing effect of 8 of 12 mutant variants and revealed the negative or neutral effects of 4 of them (3). AggreProt was capable of successfully identifying the mutational effect of 6 out of 8 solubilied variants (**Supplementary Figure S3**, C), however, it failed to predict the negative effect of the mutation H53R and T100R. Notably, the authors stated, that the arginine residues in the antibody complementarity-determining regions (CDRs) may reduce its specificity, and thus the aggregation measured using CIC showed increased non-specific interactions (3). Therefore, such an effect represents a dramatically challenging task for any predictor, which is not specifically tuned for antibodies.

Supplementary Figure S1

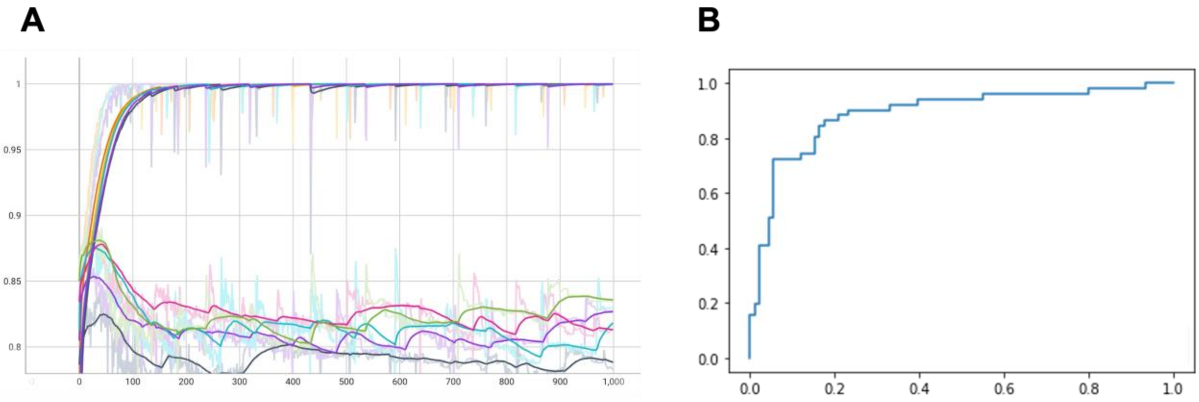

Figure S1. **AggreProt DNNs training, validation, and testing**.

A: Results of the training and validation procedure (hyperparameter optimisation) of the DNNs constituting the core of AgreProt predictor on WaltzDB-training set. The data series corresponding to each of the data splits (20:80) during the cross-validation procedure are shown in different colours. On the x-axis the training epoch is displayed, and on the y-axis the AuROC corresponding to the state of each model. The upper lines correspond to the training procedure (max(AuROCC) = 1) and the bottom ones correspond to the validation procedure, with the 20% of the data that was not used for training (max(AuROCC) = 0.890). **B**: Average results of the test procedure on the ensemble of best DNNs from the hyperparameter optimisation step on WaltzDB-testing (10% of hexapeptide data not used to train the models). The averaged AuROCC of the ensemble is 0.887, indicating that the models were not over-fitted to the data used for learning. **Note** the different scales used on the y-axis in the two panels. While in panel A it spans from 0.8 to 1.0, in panel B the range is from 0 to 1.

Supplementary Figure S2

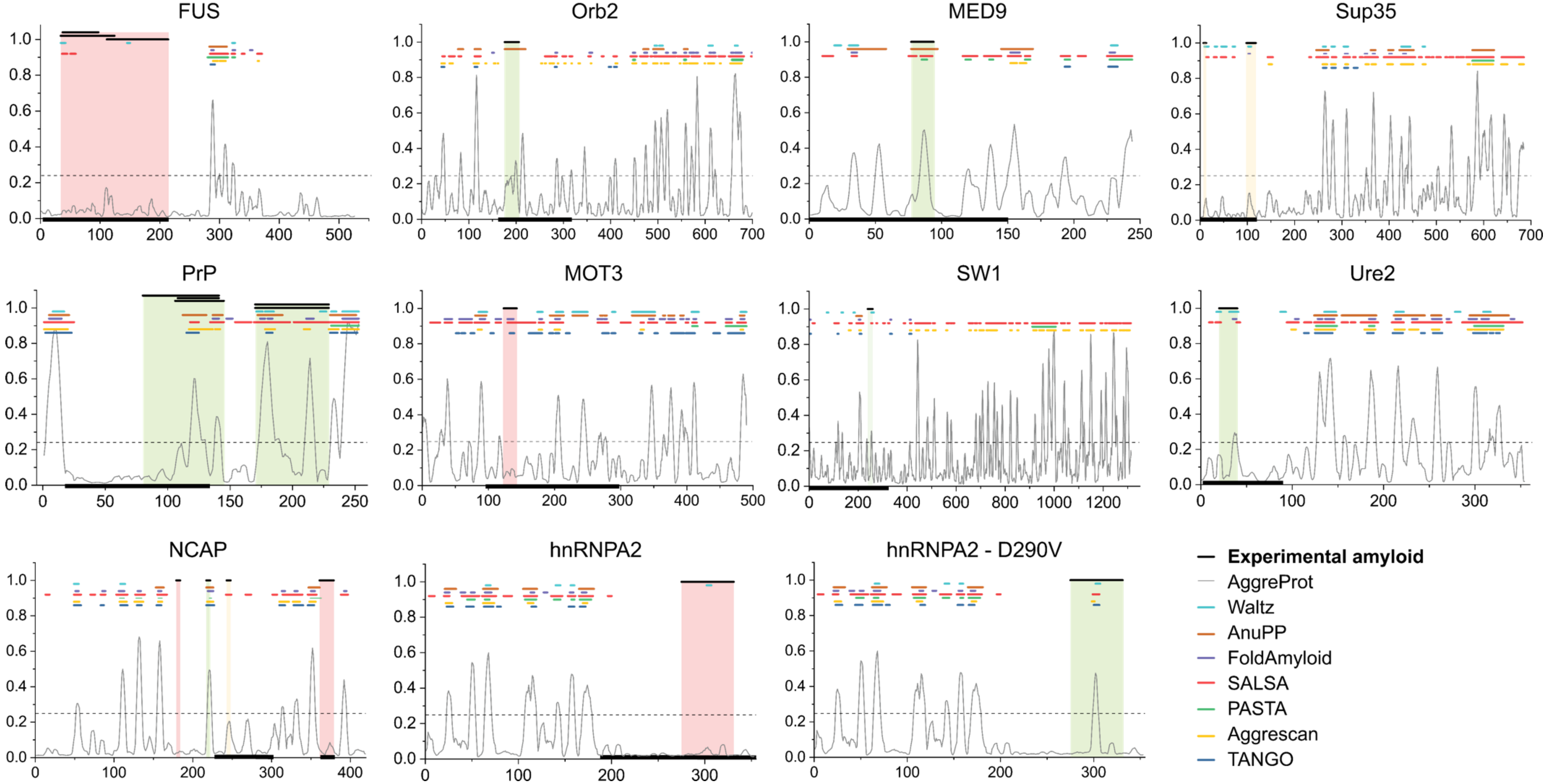

Supplementary Figure S2: AggreProt analysis of selected proteins containing low complexity domains (LCD) and prion-like domains (PrLD). The y-axis shows AggreProt score depicted by the grey lines. LCDs (FUS, NCAP, hnRNPA2, hnRNPA2-D290V) and PrLD (Orb2, MED9, Sup35, PrP, MOT3, SW1, Ure2) segments are indicated by the thick black lines on the bottom of each graph.

The segments experimentally determined to form amyloids are highlighted by black lines on top of each graph. Cases when AggreProt detects APR (score above 0.25 threshold, dotted horizontal line) in the experimentally determined amyloids are highlighted in green boxes, cases where peaks bellow threshold are detected are highlighted by yellow boxes, and no or small aggregation scores are shown as red boxes. APR prediction from other algorithms are depicted by the bars in the upper section of each graph and are color-coded according to the legend. FUS - RNA-binding protein FUS (UniProt ID: P35637, (4–6)), Orb2 - Translational regulator orb2 from Drosophila melanogaster (UniProt ID: Q9VSR3, (7)), MED9 - Mediator of RNA polymerase II transcription subunit 9 from Arabidopsis thaliana (UniProt ID: Q8RWA2, (8)), Sup35 - Eukaryotic peptide chain release factor GTP-binding subunit Saccharomyces cerevisiae (UniProt ID: P05453, (9–11)), PrP - Major prion protein, Human (UniProt ID: P04156, (12–16)), MOT3 - Transcriptional activator/repressor MOT3 Saccharomyces cerevisiae (UniProt ID: P54785, (10)), Swi1 - SWI/SNF chromatin-remodeling complex subunit SWI1 Saccharomyces cerevisiae (UniProt ID: P09547, (10)), Ure2 - Transcriptional regulator URE2 Saccharomyces cerevisiae (UniProt ID: P23202, (10)), NCAP – Nucleoprotein from SARS-CoV-2 (UniProt ID: P0DTC9, (17)), HnRNPA - Heterogeneous nuclear ribonucleoproteins A2/B1 (UniProt ID: P22626, (18)).

Supplementary Figure S3:

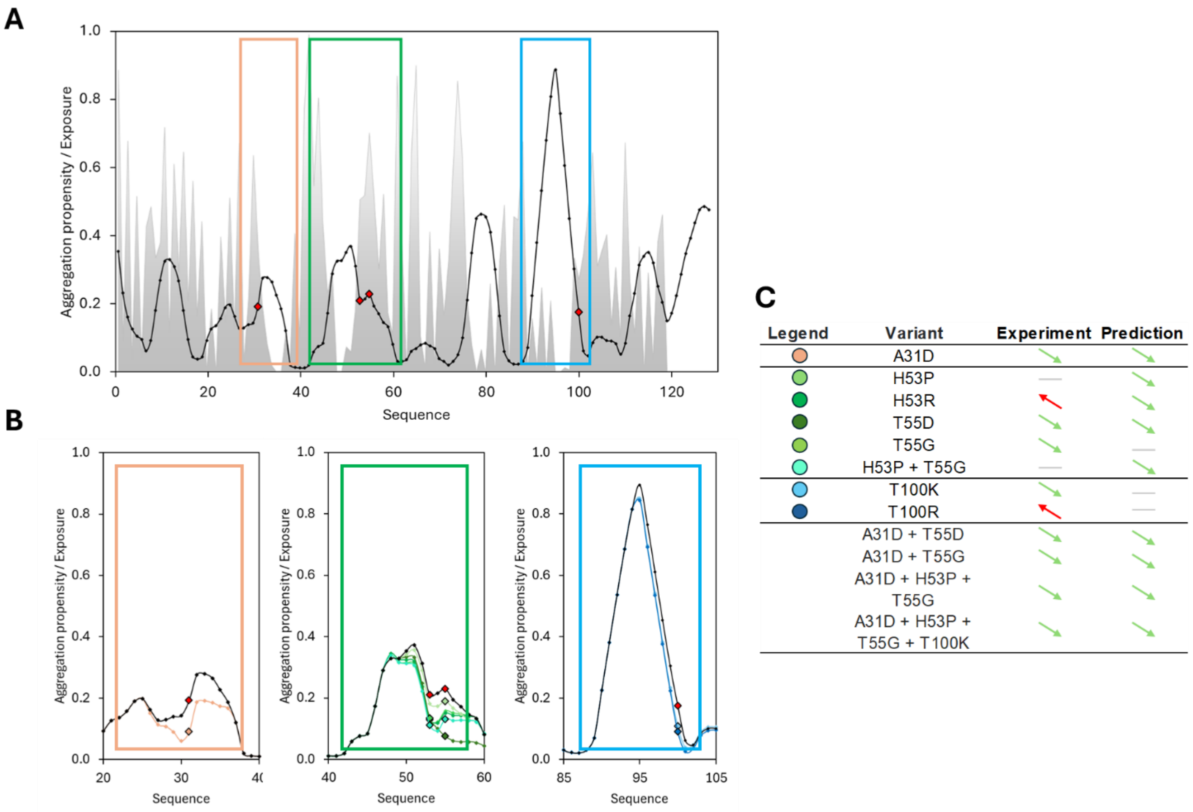

Supplementary Figure S3: The AggreProt analysis of nanobody Nb.b201 and its variants. The wild-type aggregation profile (A, black line) is depicted in the context of the surface accessibility (A, grey area) and the mutations are shown as red diamonds. The predicted effects of the respective mutations are shown as the hues in the corresponding colors (B) and the results are compared to the experimental values measured using CIC (C). The green arrow states for improved solubility, red for decreased solubility, and grey line for neutral effect.

Supplementary Table S1: Overview of the AggreProt analysis on Type III polyketide synthase mutational data. *APR* identifies the APR number (corresponding to peaks with red diamonds in **Fig 3A**. *Position* indicates the residue number where the mutation occurs. *Residue* identifies the amino acid type in the wild type sequence (wt). *Score* corresponds to the AggreProt score for that position in the wt. *Exposure* refers to the SASA value calculated for that position in the wt. *Mutation* identifies the residue type to which the position was mutated. *Location* indicates where the mutation occurs, either at the TOP of the peak, or to one of its SIDEs. *New score* indicates the AggreProt score for that position in the mutant. *Difference* is the result of subtracting *New score* from *Score*; the lower, the most solubilising effect for that mutation predicted by AggreProt. *Evaluation* summarises the effects predicted for the mutation; either "peak reduced" (predicting reduced aggregation), "no effect" (null to minor variation), or "peak increased" (indicating a predicted increased aggregation).

APR	Position	Residue	Score	Exposure	Mutation	Localization	New score	Difference	Evaluation
1	52	L	0.29	0.20	Y	TOP	0.26	-0.03	no effect
2	99	D	0.24	0.75	E	SIDE	0.23	-0.01	no effect
3	115	M	0.46	0.52	K	TOP	0.16	-0.3	peak reduced
4	254	D	0.22	0.61	K	SIDE	0.24	0.02	no effect
4	255	C	0.31	0.45	E	SIDE	0.18	-0.13	peak reduced
4	257	L	0.46	0.10	K	TOP	0.25	-0.21	peak reduced
4	262	R	0.14	0.51	D	SIDE	0.19	0.05	no effect
5	268	F	0.42	0.11	D	TOP	0.11	-0.31	peak reduced
5	268	F	0.42	0.11	N	TOP	0.22	-0.2	peak reduced
5	268	F	0.42	0.11	Q	TOP	0.24	-0.18	peak reduced
5	269	H	0.42	0.37	D	TOP	0.07	-0.35	peak reduced
5	270	C	0.35	0.19	E	TOP	0.12	-0.23	peak reduced
5	270	C	0.35	0.19	T	TOP	0.22	-0.13	peak reduced
5	270	C	0.35	0.19	N	TOP	0.19	-0.16	peak reduced
6	296	S	0.04	0.71	Y	SIDE	0.18	0.14	peak increased
7	309	G	0.25	0.10	E	SIDE	0.11	-0.14	peak reduced
7	318	S	0.1	0.64	M	SIDE	0.21	0.11	peak increased
8	341	S	0.23	0.00	N	SIDE	0.21	-0.02	no effect
8	345	V	0.8	0.00	Q	TOP	0.57	-0.23	peak reduced
8	347	F	0.81	0.03	E	TOP	0.56	-0.25	peak reduced
8	347	F	0.81	0.03	Q	TOP	0.7	-0.11	peak reduced
9	370	L	0.31	0.10	Q	SIDE	0.21	-0.1	peak reduced
9	374	L	0.5	0.00	P	TOP	0.11	-0.39	peak reduced
9	376	F	0.28	0.02	P	SIDE	0.13	-0.15	peak reduced
10	385	V	0.49	0.00	K	TOP	0.1	-0.39	peak reduced
10	385	V	0.49	0.00	N	TOP	0.12	-0.37	peak reduced
10	387	L	0.38	0.00	K	TOP	0.16	-0.22	peak reduced
10	387	L	0.38	0.00	D	TOP	0.16	-0.22	peak reduced

Supplementary Table S2: Overview of the AggreProt analysis of β-lactamase. Legend as in Supplementary Table S1. The reference aggregation profile is shown in **Figure 3C**.

APR	Position	Residue	Score	Exposure	Mutation	Localization	New score	Difference	Evaluation
1	42	V	0.19	0.02	M	SIDE	0.13	-0.06	peak reduced
1	42	V	0.19	0.02	W	SIDE	0.17	-0.02	no effect
1	46	E	0.3	0.01	L	TOP	0.6	0.3	peak increased
1	46	E	0.3	0.01	Q	TOP	0.44	0.14	peak increased
1	47	L	0.28	0	M	TOP	0.23	-0.05	peak reduced
2	67	M	0.07	0	G	SIDE	0.05	-0.02	no effect
2	69	T	0.19	0	D	SIDE	0.11	-0.08	no effect
2	70	F	0.31	0	W	SIDE	0.3	-0.01	no effect
2	71	K	0.42	0	A	TOP	0.51	0.09	peak increased
2	71	K	0.42	0	D	TOP	0.45	0.03	no effect
2	71	K	0.42	0	F	TOP	0.73	0.31	peak increased
2	71	K	0.42	0	G	TOP	0.61	0.19	peak increased
2	71	K	0.42	0	H	TOP	0.6	0.18	peak increased
2	71	K	0.42	0	N	TOP	0.6	0.18	peak increased
2	71	K	0.42	0	P	TOP	0.44	0.02	no effect
2	71	K	0.42	0	S	TOP	0.6	0.18	peak increased
2	71	K	0.42	0	Y	TOP	0.74	0.32	peak increased
2	71	K	0.42	0	E	TOP	0.49	0.07	no effect
2	76	G	0.76	0	I	TOP	0.83	0.07	no effect
3	137	L	0.55	0	Y	TOP	0.52	-0.03	no effect
4	241	R	0.08	0.07	D	SIDE	0.19	0.11	peak increased
4	242	G	0.23	0	D	SIDE	0.16	-0.07	peak reduced
5	260	Y	0.65	0	L	TOP	0.47	-0.18	peak reduced
6	278	I	0.24	0.01	P	TOP	0.03	-0.21	peak reduced
6	278	I	0.24	0.01	D	TOP	0.03	-0.21	peak reduced
6	278	I	0.24	0.01	E	TOP	0.04	-0.2	peak reduced
6	278	I	0.24	0.01	G	TOP	0.05	-0.19	peak reduced
6	278	I	0.24	0.01	H	TOP	0.16	-0.08	peak reduced
6	278	I	0.24	0.01	N	TOP	0.05	-0.19	peak reduced
6	278	I	0.24	0.01	S	TOP	0.07	-0.17	peak reduced
6	279	G	0.25	0.01	D	TOP	0.11	-0.14	peak reduced
6	281	S	0.22	0.11	P	TOP	0.14	-0.08	peak reduced
6	282	L	0.25	0.02	D	TOP	0.07	-0.18	peak reduced
6	282	L	0.25	0.02	E	TOP	0.1	-0.15	peak reduced
6	282	L	0.25	0.02	G	TOP	0.13	-0.12	peak reduced
6	282	L	0.25	0.02	H	TOP	0.23	-0.02	no effect
6	282	L	0.25	0.02	K	TOP	0.1	-0.15	peak reduced
6	282	L	0.25	0.02	N	TOP	0.14	-0.11	peak reduced
6	282	L	0.25	0.02	P	TOP	0.08	-0.17	peak reduced
6	282	L	0.25	0.02	Q	TOP	0.12	-0.13	peak reduced
6	282	L	0.25	0.02	R	TOP	0.07	-0.18	peak reduced
6	282	L	0.25	0.02	S	TOP	0.14	-0.11	peak reduced
6	282	L	0.25	0.02	T	TOP	0.17	-0.08	peak reduced
6	283	I	0.29	0.14	G	TOP	0.09	-0.2	peak reduced
6	283	I	0.29	0.14	K	TOP	0.1	-0.19	peak reduced
6	283	I	0.29	0.14	P	TOP	0.07	-0.22	peak reduced
6	283	I	0.29	0.14	Q	TOP	0.1	-0.19	peak reduced
6	283	I	0.29	0.14	D	TOP	0.05	-0.24	peak reduced
6	283	I	0.29	0.14	E	TOP	0.11	-0.18	peak reduced
6	283	I	0.29	0.14	H	TOP	0.26	-0.03	no effect
6	283	I	0.29	0.14	N	TOP	0.13	-0.16	peak reduced
6	283	I	0.29	0.14	R	TOP	0.06	-0.23	peak reduced
6	283	I	0.29	0.14	S	TOP	0.11	-0.18	peak reduced
6	283	I	0.29	0.14	T	TOP	0.13	-0.16	peak reduced
6	284	K	0.23	0.76	F	TOP	0.69	0.46	peak increased
6	284	K	0.23	0.76	P	TOP	0.21	-0.02	no effect
6	284	K	0.23	0.76	W	TOP	0.66	0.43	peak increased
6	284	K	0.23	0.76	Y	TOP	0.7	0.47	peak increased
6	284	K	0.23		G	TOP	0.4	0.17	peak increased

Supplementary File 1. AmyPro27. Collection of sequences and AmyPro annotations on the 27 proteins used for independent test. Proteins in AmyPro 27 did not contain any hexapeptide using during the traning and validation procedure.

Supplementary File 2. Validation of AggreProt on AmyPro27. Zip archive file with the results of the validation of AggreProt on individual proteins in AmyPro27. Each protein validation is contained in one *.png file, which can be identified by AmyPro ID and the protein common name. Each file is composed of three panels. On the top are shown the resulting Receiver Operatic Characteristic (ROC, left) and Precision-Recall (PRC, right) curves. On the bottom, the aggregation propensity profile as predicted by AggreProt (orange continuous line), in the context of SASA evaluated from the corresponding structure (blue line) and the ground truth annotated by AmyProt (red shaded areas for annotated APRs).

Supplementary Files 3-11. Validation of other predictors on AmyPro27. Zip archive file with the results of the validation of third-party predictors on individual proteins in AmyPro27. Each protein validation is contained in one *.png file, which can be identified by AmyPro ID and/or the protein common name. The contents of the individual files within the archive are per description in **Supplementary File 2**. The included predictors are: Aggrescan (**Supplementary File 3**), Aggrescan3D (**Supplementary File 4**), ANupp (**Supplementary File 5**), CamSol intrinsic (**Supplementary File 6**), FoldAmyloid (**Supplementary File 7**), PASTA (**Supplementary File 8**), SALSA (**Supplementary File 9**), Tango (**Supplementary File 10**), and Waltz (**Supplementary File 11**)

References

1. Lee,J., Cho,H. and Kwon,I. (2022) Phase separation of low-complexity domains in cellular function and disease. *Exp Mol Med*, **54**, 1412–1422.
https://doi.org/10.1038/s12276-022-00857-2
https://pubmed.ncbi.nlm.nih.gov/36175485/
https://www.ncbi.nlm.nih.gov/pmc/articles/PMC9534829/
2. Murray,K.A., Hughes,M.P., Hu,C.J., Sawaya,M.R., Salwinski,L., Pan,H., French,S.W., Seidler,P.M. and Eisenberg,D.S. (2022) Identifying amyloid-related diseases by mapping mutations in low-complexity protein domains to pathologies. *Nat Struct Mol Biol*, **29**, 529–536.
https://doi.org/10.1038/s41594-022-00774-y
https://pubmed.ncbi.nlm.nih.gov/35637421/
https://www.ncbi.nlm.nih.gov/pmc/articles/PMC9205782/
3. Rosace,A., Bennett,A., Oeller,M., Mortensen,M.M., Sakhnini,L., Lorenzen,N., Poulsen,C. and Sormanni,P. (2023) Automated optimisation of solubility and conformational stability of antibodies and proteins. *Nat Commun*, **14**, 1937.
https://doi.org/10.1038/s41467-023-37668-6
https://pubmed.ncbi.nlm.nih.gov/37024501/
https://www.ncbi.nlm.nih.gov/pmc/articles/PMC10079162/
4. Lee,M., Ghosh,U., Thurber,K.R., Kato,M. and Tycko,R. (2020) Molecular structure and interactions within amyloid-like fibrils formed by a low-complexity protein sequence from FUS. *Nat Commun*, **11**, 5735.
https://doi.org/10.1038/s41467-020-19512-3
https://pubmed.ncbi.nlm.nih.gov/33184287/
https://www.ncbi.nlm.nih.gov/pmc/articles/PMC7665218/

5. Sun,Y., Zhang,S., Hu,J., Tao,Y., Xia,W., Gu,J., Li,Y., Cao,Q., Li,D. and Liu,C. (2022) Molecular structure of an amyloid fibril formed by FUS low-complexity domain. *iScience*, **25**, 103701.
https://doi.org/10.1016/j.isci.2021.103701
https://pubmed.ncbi.nlm.nih.gov/35036880/
https://www.ncbi.nlm.nih.gov/pmc/articles/PMC8749265/
6. Murray,D.T., Kato,M., Lin,Y., Thurber,K.R., Hung,I., McKnight,S.L. and Tycko,R. (2017) Structure of FUS Protein Fibrils and Its Relevance to Self-Assembly and Phase Separation of Low-Complexity Domains. *Cell*, **171**, 615-627.e16.
https://doi.org/10.1016/j.cell.2017.08.048
https://pubmed.ncbi.nlm.nih.gov/28942918/
https://www.ncbi.nlm.nih.gov/pmc/articles/PMC5650524/
7. Hervas,R., Rau,M.J., Park,Y., Zhang,W., Murzin,A.G., Fitzpatrick,J.A.J., Scheres,S.H.W. and Si,K. (2020) Cryo-EM structure of a neuronal functional amyloid implicated in memory persistence in *Drosophila*. *Science (1979)*, **367**, 1230–1234.
https://doi.org/10.1126/science.aba3526
https://pubmed.ncbi.nlm.nih.gov/32165583/
https://www.ncbi.nlm.nih.gov/pmc/articles/PMC7182444/
8. Pintado-Grima,C., Santos,J., Iglesias,V., Manglano-Artuñedo,Z., Pallarès,I. and Ventura,S. (2023) Exploring cryptic amyloidogenic regions in prion-like proteins from plants. *Front Plant Sci*, **13**.
https://doi.org/10.3389/fpls.2022.1060410
https://pubmed.ncbi.nlm.nih.gov/36726678/
https://www.ncbi.nlm.nih.gov/pmc/articles/PMC9885169/
9. Sawaya,M.R., Rodriguez,J., Cascio,D., Collazo,M.J., Shi,D., Reyes,F.E., Hattne,J., Gonen,T. and Eisenberg,D.S. (2016) Ab initio structure determination from prion nanocrystals at atomic resolution by MicroED. *Proceedings of the National Academy of Sciences*, **113**, 11232–11236.
https://doi.org/10.1073/pnas.1606287113
https://pubmed.ncbi.nlm.nih.gov/27647903/
https://www.ncbi.nlm.nih.gov/pmc/articles/PMC5056061/
10. Sant'Anna,R., Fernández,M.R., Batlle,C., Navarro,S., de Groot,N.S., Serpell,L. and Ventura,S. (2016) Characterization of Amyloid Cores in Prion Domains. *Sci Rep*, **6**, 34274.
https://doi.org/10.1038/srep34274
https://pubmed.ncbi.nlm.nih.gov/27686217/
https://www.ncbi.nlm.nih.gov/pmc/articles/PMC5043269/
11. Toombs,J.A., Liss,N.M., Cobble,K.R., Ben-Musa,Z. and Ross,E.D. (2011) [PSI+] Maintenance Is Dependent on the Composition, Not Primary Sequence, of the Oligopeptide Repeat Domain. *PLoS One*, **6**, e21953.
https://doi.org/10.1371/journal.pone.0021953
https://pubmed.ncbi.nlm.nih.gov/21760933/
https://www.ncbi.nlm.nih.gov/pmc/articles/PMC3132755/
12. Wang,L.-Q., Zhao,K., Yuan,H.-Y., Wang,Q., Guan,Z., Tao,J., Li,X.-N., Sun,Y., Yi,C.-W., Chen,J., *et al.* (2020) Cryo-EM structure of an amyloid fibril formed by full-length human prion protein. *Nat Struct Mol Biol*, **27**, 598–602.
https://doi.org/10.1038/s41594-020-0441-5
https://pubmed.ncbi.nlm.nih.gov/32514176/
13. Glynn,C., Sawaya,M.R., Ge,P., Gallagher-Jones,M., Short,C.W., Bowman,R., Apostol,M., Zhou,Z.H., Eisenberg,D.S. and Rodriguez,J.A. (2020) Cryo-EM structure of

a human prion fibril with a hydrophobic, protease-resistant core. *Nat Struct Mol Biol*, **27**, 417–423.
https://doi.org/10.1038/s41594-020-0403-y
https://pubmed.ncbi.nlm.nih.gov/32284600/
https://www.ncbi.nlm.nih.gov/pmc/articles/PMC7338044/
14. Li,Q., Jaroniec,C.P. and Surewicz,W.K. (2022) Cryo-EM structure of disease-related prion fibrils provides insights into seeding barriers. *Nat Struct Mol Biol*, **29**, 962–965.
https://doi.org/10.1038/s41594-022-00833-4
https://pubmed.ncbi.nlm.nih.gov/36097290/
https://www.ncbi.nlm.nih.gov/pmc/articles/PMC9639217/
15. Hallinan,G.I., Ozcan,K.A., Hoq,M.R., Cracco,L., Vago,F.S., Bharath,S.R., Li,D., Jacobsen,M., Doud,E.H., Mosley,A.L., *et al.* (2022) Cryo-EM structures of prion protein filaments from Gerstmann–Sträussler–Scheinker disease. *Acta Neuropathol*, **144**, 509–520.
https://doi.org/10.1007/s00401-022-02461-0
https://pubmed.ncbi.nlm.nih.gov/35819518/
https://www.ncbi.nlm.nih.gov/pmc/articles/PMC9381446/
16. Watts,J.C., Bourkas,M.E.C. and Arshad,H. (2018) The function of the cellular prion protein in health and disease. *Acta Neuropathol*, **135**, 159–178.
https://doi.org/10.1007/s00401-017-1790-y
https://pubmed.ncbi.nlm.nih.gov/29151170/
17. Tayeb-Fligelman,E., Bowler,J.T., Tai,C.E., Sawaya,M.R., Jiang,Y.X., Garcia,G., Griner,S.L., Cheng,X., Salwinski,L., Lutter,L., *et al.* (2023) Low complexity domains of the nucleocapsid protein of SARS-CoV-2 form amyloid fibrils. *Nat Commun*, **14**, 2379.
https://doi.org/10.1038/s41467-023-37865-3
https://pubmed.ncbi.nlm.nih.gov/37185252/
https://www.ncbi.nlm.nih.gov/pmc/articles/PMC10127185/
18. Lu,J., Cao,Q., Hughes,M.P., Sawaya,M.R., Boyer,D.R., Cascio,D. and Eisenberg,D.S. (2020) CryoEM structure of the low-complexity domain of hnRNPA2 and its conversion to pathogenic amyloid. *Nat Commun*, **11**, 4090.
https://doi.org/10.1038/s41467-020-17905-y
https://pubmed.ncbi.nlm.nih.gov/32796831/
https://www.ncbi.nlm.nih.gov/pmc/articles/PMC7427792/
